# Characteristics of Italian, German and Spanish Socio-Economic, Public Health and Long-Term Care Systems Associated with COVID-19 Incidence and Mortality in the First Pandemic Year: Lessons for Future Sustainability in an International Perspective

**DOI:** 10.3390/healthcare12192006

**Published:** 2024-10-08

**Authors:** Georgia Casanova, Roberto Lillini, Giovanni Lamura

**Affiliations:** 1 Centre for Socio-Economic Research on Ageing, IRCCS-INRCA National Institute of Health and Science on Ageing, 60124 Ancona, Italy; g.casanova@inrca.it (G.C.); g.lamura@inrca.it (G.L.); 2 Data Science Unit, Department of Epidemiology and Data Science, IRCCS National Cancer Institute Foundation (INT), 20133 Milan, Italy

**Keywords:** ageing, long-term care, COVID-19, sustainability, socio-economic conditions, health and social care systems

## Abstract

**Background/Objectives:** The main outcomes of the COVID-19 pandemic can be used to assess the capability and sustainability of public healthcare and Long-Term Care (LTC) systems. This study aims to identify the population’s demographic and socio-economic characteristics, as well as other national resources associated with the incidence and mortality of COVID-19, by comparing three European countries during the first pandemic period (Italy, Spain, and Germany). The results will identify possible strengths and weaknesses that could be considered as hints of the need for health and social intervention. **Methods:** Variables describing the countries’ core demographics, socio-economic characteristics, and national resources were collected from 2001–2021 from well-established international databases. COVID-19 incidence and death figures from 1 March 2020 to 31 March 2021 were extracted from national health databases. Analysis focused on bivariate and weighted multivariable linear regressions between incidence, mortality, and socio-economic covariates. **Results:** Findings show that both care models and socio-demographic characteristics influenced the capability of the first year’s response to the COVID-19 emergency. Formal public care appears to represent the most effective strategy against incidence and mortality regarding COVID-19, especially for older people, because it mitigates the adverse effects of socio-economic characteristics. **Conclusions:** Current strategies oriented towards privatizing care should, therefore, be considered critically, since they may result in weaker protection of vulnerable groups, such as frail older people, due to the unequal position of individuals with different socio-economic conditions in purchasing services from the care market.

## 1. Introduction

The ageing of the population is now a notable phenomenon that significantly influences Western societies. By 2050, the number of people aged 60 and older will nearly double to make up 22% of the total population, while the number of people aged 80 and older will likely quadruple [1]. These ageing trends bring attention to the growing care needs that Long-Term Care (LTC) systems are called upon to respond to [2].

The 2008–2009 European economic crisis severely hit several nations’ care systems, which led to reductions and layoffs in long-term care [3]. Furthermore, the COVID-19 pandemic has severely strained public health systems worldwide, requiring significant investments and changing national budgets in numerous nations [4]. The literature shows a growing interest in finding elements to support new sustainability for European LTC [5,6]. Cross-country studies can help find successful policy solutions in this context.

Health crises, like the COVID-19 pandemic, are crucial litmus tests for the viability and capability of welfare programs implemented across various sectors. [7,8]. In particular, the management of COVID-19 had a direct connection with the long-term care sector because frail older people meet a higher risk of infection and mortality [9] and because older people living in facilities have been strongly affected by COVID-19 in many European countries [10]. Recent literature has primarily focused on COVID-19′s repercussions at the micro-level of care to understand its effect on care recipients and professionals in care facilities [11,12,13,14] The literature still scarcely discusses the macro point of view of the effect of the COVID-19 pandemic on the LTC system.

In 2022, the OECD highlighted how discrepancies between countries in mortality associated with COVID-19 are due to differences in the age structure of their populations and in the strategies adopted to contain the virus, as well as the different capacities of health systems to manage the patients affected by COVID-19 [15]. This study aims to contribute to the debate on the LTC system’s ability to answer to a health emergency by offering a pilot macro-level analysis in three different European countries selected for the direct impact of COVID-19 on their older population and for a study of the implementation of dissimilar strategies during the COVID-19 pandemic. Italy, Spain and Germany correspond to these characteristics. Indeed, OECD data emphasizes how, in these three countries, mortality from COVID-19 was higher than in other European countries [15].

Rocard and colleagues [16] analyzed 19 OECD countries and highlighted how the strategies to counteract the pandemic’s spread and effects were characterized by some differences, according to the chosen national strategy. In 2020, Germany applied many policy measures to protect LTC recipients and workers from COVID-19 and maintain continuity of care during the crisis, including improving the regulatory regime for informal care. Italy and Spain applied most of the standard measures suggested by supranational organizations to maintain distancing and implement vaccinations, applying them to the LTC sector regarding restrictions in facilities’ care, preventative test procedures and guidelines for better services coordination and services, or access to services. However, in these Mediterranean countries, the implementation of measures has been firmly determined by the adopted multilevel governance of the health and LTC sectors [17]. In both countries, lockdown periods were specified at the national level. However, at the local level, each Italian Region and Spanish Autonomous Community defined and implemented specific strategies based on national guidelines promoted by the Ministries. 

With regard to the countries’ characteristic frameworks, Italy, Spain, and Germany represent two of Europe’s different existing care regimes (family-based care and mixed care regimes), based on divergent care demand levels, various types of care services and disparate balances between formal and informal care provisions [18] According to this, such countries demonstrate dissimilar strategies for LTC spending, the use of public or private resources, and different LTC system governance, including countries with regional or local governance (Italy, Spain) and national LTC models (Germany) [19,20].

This study aims to discover these populations’ demographic and socio-economic characteristics and explore various aspects of national resources associated with the incidence and mortality of COVID-19 during the first year, comparing three European countries: Italy, Spain, and Germany.

A second aim is to discover if the characteristics discovered show significant differences between the three countries, to stress possible strengths and weaknesses that could be considered intervention factors regarding the different health and social policies.

Despite the weaknesses related to pilot studies, this study contributes to the literature, supporting a better understanding and helping formulate evidence-based suggestions for the future sustainability of international LTC systems.

## 2. Materials and Methods

Variables describing the main population demographics and socio-economic characteristics and national resources for Italy, Germany and Spain (the covariates) were collected for the period 2001–2021 from the main international, freely available databases [21,22]. They regarded the following areas: Home care, Residential care, Health Expenditure, Coverage of services, Cash benefits, Private services, Population, Family structure, Education level, Employment, Poverty, Disability and care, Life expectancy.

All the considered 52 variables were measured as percentages or other normalized rates, in order to allow reliable comparison between countries [23]. The criteria by which such variables were grouped and treated answered to methods and techniques already applied in and shared with other studies at an international/cross-country level according to established works from other authors [24,25,26] and from the authors of this study [27,28,29,30]. All of these studies were aimed at finding and describing the association between the characteristics of the population and the national systems and their capabilities in answering to the health and social needs of their people, by considering the information supplied to international consolidated databases (e.g., Eurostat, WHO, OECD).

The complete list of the variables is reported in Table 1.

COVID-19 incidence and death figures (the dependent variables) were collected for the period 1 March 2020–31 March 2021 from the health databases of the national statistics office. They were considered as absolute variables and rates (per million population or percentage) (Table 2).

The average values of both target variables and covariates were considered after checking that no outliers were present along their distribution in the considered time period [31].

The synthesized variables were used in the following analyses in three steps, according to the procedures applied in Casanova et al. (2024), which similarly involved national demographic, socio-economic and health and social systems-related variables to describe the association between health outcomes and such national descriptors [23].

(1) A Pearson’s bivariate correlation analysis (statistical significance threshold at *p* < 0.05) between all the covariates and the target variables was performed, in order to select only the descriptors associated with incidence and mortality according to their statistical significance [32].
(2) A weighted multivariable linear regression analysis between the various target variables and the corresponding covariates previously selected allowed computing of the Relative Risk Ratios (RRR) based on the obtained beta coefficients (statistical significance at *p* < 0.05; collinearity test by tolerance and VIF at *p* < 0.001) [32,33].
(3) Comparison was made of the statistically significant covariate levels found in the regression analysis for the three nations to evaluate the relevance of the strengths and weaknesses of Italy with respect to the other countries. Pairwise, *t*-test at *p* < 0.05 was applied to verify if the differences between Italy, Germany and Spain were statistically significant [32,33].

In order to evaluate the effects of old age, the analyses were performed on an all-age population, 70+ years old and 80+ years old people for both incidence and mortality.

All the analyses were performed by SPSS 19.0 (IBM, Armonk, NY, USA) and Stata 17.0 (StataCorp LLC, College Station, TX, USA) statistical software [34,35].

## 3. Results

Table 3 and Table 4 report the bivariate selection of the variables with respect to incidence and mortality, stressing a strong selection from the original high number of considered covariates.

In Table 3 the variables which are positively and negatively associated with incidence are shown. Such variables are a first indication of what elements can be considered a strength (negative association, therefore a contribution to reducing the incidence) or a weakness (positive association, a contribution to increasing the incidence) for the national health and social systems. Similarly, variables in Table 4 supply the same information related to mortality.

Only three variables remarked a statistically significant negative correlation with incidence: Six-and-more person families for total population, Hospital admission per million for 70+ years old population, and Care workers for the elderly in structure (%) for 80+ years old population.

More variables presented a statistically significant positive correlation with incidence: Ageing Index—F, Industry employment (%), Poor persons—All (%), Severe materially deprived—Unemployed (%), Elderly care health facilities rate (% on 65+ pop.) when considering the total population; Severe materially deprived—Retired (%), Severe materially deprived—Others outside labor force (%), Disability Rate—65+ M (% on 65+ M) for 70+ years old people; Severe materially deprived—lower secondary education level (%) among 80+ years old persons.

With respect to mortality, a statistically significant negative correlation was found for Private Health Expenditure—% of the GDP on total population, % of population with postsecondary education aged 25+ year and Active Population—M% on 70+ years old people, % of population with postsecondary education aged 25+ year and Active Population—M% on 80+ years old persons.

Again, more covariates showed statistically significant positive correlation with mortality: Two-person poor households (%), Disability Rate—15–64 F (% on 15–64 F), Disability Rate—15+ F (% on 15+ F), Disability rates in activities of daily living (ADL) (%)—55–64 M, Disability rates in activities of daily living (ADL) (%)—65+ M on total population; Dependency Ratio (%)—M and Disability rates in activities of daily living (ADL) (%)—55+ M on 70+ years old people; Dependency Ratio (%)—M, Two-person poor households (%) and Disability rates in activities of daily living (ADL) (%)—55+ M on 80+ years old persons.

The above covariates were inserted in the multivariable regression models, whose result are shown in Table 5 and Table 6. Average values of the statistically significant covariates in the model and the statistical significance of the differences among countries by Pairwise *t*-test are presented in Table 7.

Such results improve the identification of the relevant variables that characterize the strengths and weakness of the considered countries with respect to the ability of their health and social systems in having dealt with the COVID-19 pandemic, when these covariates are considered together in a model. RRR coefficients help to quantify the impact on the association of the variables which remains after the collinearity check.

Moreover, the results in Table 7 stress the existence of differences between countries, despite their similarities.

Considering Incidence (Table 5), Ageing Index—F and Elderly care health facilities rate (% on 65+ pop.) were the only significant covariates (RRR = 1.65 for both), remarking an increasing in incidence risk. The stochastic variable was protective.

Elderly care health facilities rates were higher in Italy and Germany than in Spain, thus more relevant for increasing incidence (the difference was statistically significant by Pairwise *t*-test with *p* < 0.05). No statistically significant difference was recorded for Ageing Index—F (*p* > 0.05), thus its relevance was similar for the three countries.

For 70+ people, Severe materially deprived—Others outside labor force (%) increased incidence risk (RRR = 1.37), while Hospital admission per million reduced the risk (RRR = 0.50). Severe materially deprived—Others outside labor force is particularly relevant in Italy, which reported the highest value, with fewer effects in the other countries. Hospital admission per million was a weakness for Italy, which showed the lowest value, but not for Germany and Spain, which were similar. Differences among countries were statistically significant (Pairwise *t*-test, *p* < 0.05).

For very old people (80+), both Severe materially deprived—lower secondary education level (%) (RRR = 8.93) and Care workers for the elderly in structure (%) (RRR = 3.29) increased the risks of incidence, while the stochastic variable reduced them. Severe materially deprived—lower secondary education level was higher and relevant in Italy, similar to Germany and different from Spain. Such differences among countries were statistically significant (*p* < 0.05). There were no real difference among countries for Care workers for the elderly in structure.

Considering Mortality (Table 6), Two-person poor households (%) increased risks (RRR = 1.54), while Private Health Expenditure—% of the GDP was protective (RRR = 0.57). Two-person poor households was less relevant in Italy than in Spain or Germany. There were no statistically significant differences for Private Health Expenditure among countries.

For 70+ people, Dependency Ratio (%)—M and Disability rates in activities of daily living (ADL) (%)—55+ M increased incidence risk (RRR = 2.98 and 1.10, respectively). The stochastic variable was protective. Disability rates in activities of daily living (ADL) (%)—55+ M was more relevant in Italy than in Spain or Germany, which showed slight differences between them. No significant difference among countries for Dependency Ratio (%)—M was recorded.

For very old people (80+), the same variables as for 70+ people were found, with the same effects. Only RRR changed (1.34 and 2.04, respectively).

## 4. Discussion

The bivariate analysis results support the study’s conceptual framework, because they corroborate the existing literature in terms of impact of the LTC system approach on the incidence of COVID-19 and related mortality in the population [15], especially with regard to accessibility to formal and informal care [36]. In particular, the availability of care workers for the elderly, the Elderly care health facilities rate and the incidence of hospital admissions support the protective effect of formal care. At the same time, the negative correlation between the share of large families and the incidence of contagion underlines the protection effect of informal care. The presence of Elderly care health facilities and Care workers for the elderly in structure rates both seem to contribute to an increase in contagious incidence risk. This result draws attention to the effect of the national containment and distancing policies applied during the COVID-19 emergency, as remarked by the OECD [15].

Furthermore, the bivariate results show that living in deprived conditions, in terms of poverty, low education or unemployment, increases the risk of incidence and mortality related to COVID-19, confirming the evidence highlighted by the literature with regard to the impact of socio-economic deprivation on the risk of illness for frail people, especially in older age [37,38]. In comparative terms, the similarities detected between Germany and Italy on the effect of Elderly care health facilities’ rates, higher than Spain, stressed the role of the care strategies implemented in these countries. According to the data, in Italy a care strategy closer to the German approach has been embraced, rather than to the Spanish example. This confirms that the Italian LTC system is multi-faceted and has some elements of a mixed care regime, as indicated by the literature [39], and that residential care should be given attention to in improving this care strategy. Differently, Spanish data seem to reflect a strategy based on available local services, informal care, and the individual responsibility of citizens [40]. In compliance with the relevance of residential care as part of the LTC system, the protective power of hospitalization admissions in terms of the incidence of COVID-19 points out that residential care should be enhanced, particularly in Italy.

Efficient Nursing homes for older people can help primary health care respond to the threats to the health needs of older people, making the difference in a successful approach in combating future health emergencies. In this framework, the statistical significance of care workers’ rates in elder care facilities for COVID-19 incidence in 80+ people shows that the care worker issue is a priority: a specialized and quantitatively robust workforce offers more elasticity in the management of staff and shift turns, offering better protection for care recipients from accidental contagion by workers [41]. In Germany and Spain, the safeguarding effect of hospitalization was better managed, positively impacting on the incidence of contagion and mortality among older people. The reason for this result should be investigated in more depth, considering the similarity between the Spanish and Italian care models, both characterized by a mainly local governance of health and LTC services. However, in Germany and Spain, the organization of local primary care services based on collaboration between local health districts and hospitals reduced the effect on hospitals, consequently allowing hospital admissions to maintain a protective effect [42,43]. On the contrary, Italian general practitioners and hospitals were the two pillars of fighting COVID-19, without the support provided by intermediate structures [44].

The hypothesis that living in conditions of deprivation, with low levels of income or education, may reduce the ability to combat the incidence of and mortality caused by COVID-19 in older people lies on the assumption that individual action for prevention and treatment is a function of personal availability of economic resources [45]. The protective effect of the rate of private health spending confirms that, in a context with a restricted provision of formal and public care, the ability of the individual or family to purchase appropriate support makes a difference between health and illness and between survival and death. In particular, in Germany and Italy, countries where health privatization strategies have historically been adopted or are spreading in recent years, people with a low education level and lower income face more difficulties in preventing contagious disease and caring about it [46,47]. In Italy, however, poor households—when composed of two people—were better able to respond to the need for care than in the other two countries studied, because supportive welfare policies better protect them [48]. 

Summarizing the leading lessons emerging from this study, the effective response of LTC systems to COVID-19 as an example of a health emergency can be influenced by the availability of (a) access to formal LTC and health services; (b) a specialized workforce for LTC services, in particular for residential facilities, to allow safe management of care paths; (c) a multilevel model of local LTC services, including residential facilities; (d) resources at individual or household level to be invested in out-of-pocket care; (e) welfare protective schemes for frail and deprived citizens, particularly older people.

The lack or insufficient presence of these conditions is likely to negatively impact the incidence of illness and mortality caused by COVID-19 in older people. 

The limits of this study are related to the fact that it focuses on only three countries, which prevents it from offering internationally broader reflections from a European perspective. Secondly, and strongly related to the first aspect, this study does not deliver intra-national, regional, and local differences, which may be equally strong or even stronger than international ones. Lastly, this study is based on national and international secondary data and, even if certified by the source, it comes with certain limitations, such as inconsistencies or incompleteness, timeliness (lack of update), heterogeneity of data (due to slight differences in definitions, collection techniques, etc.), and representativeness. Moreover, they could not include elements related to informal care and other aspects of local services provisions, which notoriously and strongly characterize the Spanish and Italian care models.

## 5. Conclusions

The study analyses and compares Italian, German, and Spanish LTC and socio-demographic contexts to identify which population characteristics and various aspects of national resources may have influenced the incidence of and mortality related to COVID-19 in the first pandemic year. This study shows that, in all three countries, the care model and socio-demographic characteristics influenced the capability of the response to the COVID-19-related health emergency. In this framework, formal and public care availability seem to stand out as the main strategy to combat incidence and mortality among older people in all three countries, because it contributes to mitigating the negative association of some socio-economic characteristics, protecting frailer older people. Conversely, strategies oriented to privatization of care, as happened in Germany and partially in Italy, result in a lower protection from the illness and mortality of older people, due to the more significant effect of individual socio-economic conditions on the ability of purchasing appropriate care schemes and/or services. The pressure on hospitals and residential facilities was determined as the main weakness of the Italian response due to the overburden of demand and low support provided by other system elements. The evidence-based suggestions from this study are relevant for the future design and implementation of sustainable LTC systems.

This pilot study suggests the development of a new analysis that includes more countries with different characteristics, in order to understand the sustainability factors of LTC from a wider perspective. Future studies should further explore international territorial differences (e.g., NUTS areas) or compare local experiences, to allow a more in-depth analysis of the effects of specific characteristics of different care models (e.g., informal care or LTC insurance schemes).

## Figures and Tables

**Table 1:** Variables collected (and verified in terms of validity and reliability) by concept areas and source database.

Issues	Indicators	Consulted Database
Home care	Pop 65+ treated in integrated home care (%)	WHO HFA-Europe
Residential care	Elderly care health facilities rate on pop 65+ (%)	WHO HFA-Europe
	Residential beds Nursing home for the elderly (a.v., %)	WHO HFA-Europe *
	Residential beds in health and social residence for the elderly (a.v., %)	Eurostat Database #
	Care workers for the elderly in structure (%)	WHO HFA-Europe *
Health Expenditure	Current public health expenditure per capita (%)	WHO HFA-Europe *
	Public health expenditure corresponded per capita in total convention for social benefits (% of GdP)	Eurostat Database #
	Total Health Expenditure (THE—US$ pp, per capita; a.v., % of GDP)	WHO HFA-Europe *
	Total government expenditure as % of GDP	WHO HFA-Europe *
	Public-sector health expenditure as % of total health expenditure and GDP	WHO HFA-Europe *
	Private-sector expenditure on health as % of total health expenditure and GDP	WHO HFA-Europe *
	Gross domestic product (GDP—US$ pp, per capita)	WHO HFA-Europe *
Coverage of services	Index of territorial coverage of the services (per 100 pop.)	WHO HFA-Europe *
Cash benefits	Number of total disability pensions	Eurostat Database #
	Average monthly amount for total disability pensions	Eurostat Database #
	Average monthly amount of accompanying allowance for total invalids	Eurostat Database #
Private services	Out-of-Pocket expenditure for health services	WHO HFA-Europe *
	Out-of-Pocket expenditure for social services	WHO HFA-Europe *
	Number of family assistants (carers) (per-100,000)	WHO HFA-Europe *
Population	Resident population by sex and age (a.v.; %)	WHO HFA-Europe
	Dependency ratio (%)	Computed by data from WHO HFA-Europe *
	Ageing index (%)	WHO HFA-Europe *
Family	Average number of components	Eurostat Database #
	Frequency of the number of components (from 1 to 6 member) (%)	Eurostat Database #
	Older people (65+ years old) living alone (%)	Eurostat Database #
Education	Literacy rate in population aged 15+ year	WHO HFA-Europe *
	% of population with postsecondary education aged 25+ year	WHO HFA-Europe *
	% of population with primary education only aged 25+ years	WHO HFA-Europe *
	% of population with secondary education only aged 25+ years	WHO HFA-Europe *
	Human Development Index	WHO HFA-Europe *
Employment	Active population rate (15–64) (%)	Eurostat Database #
	Labor force (%)	WHO HFA-Europe *
	Unemployment rate (%)	WHO HFA-Europe *
	Youth unemployment rate (15–24) (%)	WHO HFA-Europe *
	Frequency of employment in economic sectors (Industry, Agriculture, Tertiary Sector and other activities) (%)	World Bank—World Development DB §
Poverty	People at risk of poverty and social exclusion (%)	WHO HFA-Europe *
	Poor (a.v.)	Eurostat Database #
	Poor families (%)	Eurostat Database #
	Incidence of poverty (people) (%)	Computed by data from Eurostat Database #
	Frequency of poor families for no. of family members (1–6) (%)	Eurostat Database #
	Poor families with at least 1 child (%)	Eurostat Database #
	Poor families according to the structure (single-parent; with at least one child) (%)	Eurostat Database #
	Distribution of poor couples by n. of children (1–3+)	Eurostat Database #
	Severe material deprivation by age (0–64, 65+) (%)	Eurostat Database #
	Severe material deprivation by employment status (age 18+) (%)	Eurostat Database #
	Severe material deprivation by education level (age 18+) (%)	Eurostat Database #
Disability and care recipients	Disability rate (%)	WHO HFA-Europe—Eurostat Database * #
	Disability rate by age group (6–64; 65+) (%)	WHO HFA-Europe—Eurostat Database * #
	Disability rate in activities of daily living (ADL) (%)	WHO HFA-Europe—Eurostat Database * #
	Older people with ADL limitations (%)	WHO HFA-Europe—Eurostat Database * #
Life expectancy	Life expectancy in good health (yrs.)	WHO HFA-Europe—Eurostat Database * #
	Expected healthy life years at age 65 (yrs.)	WHO HFA-Europe *
COVID-19 infection health system indicators (1 January 2020–31 December 2023)	Number of cases (total)	Local National statistics Offices
	Number of deaths (total)	
	Cases per Million	
	Deaths per Million	
	Infection rate for old (70+) people (on total 70+ pop.) (%)	
	Infection rate for old (70+) people (on total COVID-19 cases) (%)	
	Infection rate for old old (80+) people (on total 80+ pop.) (%)	
	Infection rate for old old (80+) people (on total COVID-19 cases) (%)	
	Death rate for old (70+) people (on total 70+ pop.) (%)	
	Death rate for old (70+) people (on total COVID-19 cases) (%)	
	Death rate for old old (80+) people (on total 80+ pop.) (%)	
	Death rate for old old (80+) people (on total COVID-19 cases) (%)	
	COVID-19 hospital admission (total)	
	COVID-19 hospital admission per Million	

Link to database: * https://gateway.euro.who.int/en/datasets/european-health-for-all-database/ (accessed on: 12 July 2024), # https://ec.europa.eu/eurostat (accessed on: 12 July 2024), § https://data.worldbank.org/ (accessed on: 12 July 2024).

**Table 2:** Incidence and death rates by country.

	Germany	Italy	Spain
Total cases per million	449,917.37	428,197.23	287,928.34
Infections rate for older people (70+) (on total 70+ population) (%)	18.92	34.27	16.03
Infections for old old people (80+) (on total 80+ population) (%)	25.54	34.65	18.63
Total deaths per million	1951.40	3140.67	2468.81
Rate of death for older people (70+) (on total 70+ population) (%)	0.86	2.27	1.27
Rate of death for old old people (80+) (on total 80+ population) (%)	1.58	3.51	2.24

**Table 3:** Variables correlated to the COVID-19 Incidence Rates for total population (per million people) and for 70+ and 80+ years old (%).

**Effects of Reduction on Total Population Cases (per Million)**	**Pearson’s r**	**Effects of Increasing on Total Population Cases (per Million)**	**Pearson’s r**
Six-and-more person families	−0.999	Ageing Index—F	0.988
		Industry employment (%)	0.999
		Poor persons—All (%)	0.997
		Severe materially deprived—Unemployed (%)	0.999
		Elderly care health facilities rate (% on 65+ pop.)	0.998
**Effects of reduction on Infection Rate for old (70+) people (on total 70+ pop.) (%)**	**Pearson’s r**	**Effects of increasing Infection Rate for old (70+) people (on total 70+ pop.) (%)**	**Pearson’s r**
Hospital admission per million	−0.999	Severe materially deprived—Retired (%)	0.997
		Severe materially deprived—Others outside labor force (%)	0.997
		Disability Rate—65+ M (% on 65+ M)	0.999
**Effects of reduction on Infection Rate for old old (80+) people (on total 80+ pop.) (%)**	**Pearson’s r**	**Effects of increasing Infection Rate for old old (80+) people (on total 80+ pop.) (%)**	**Pearson’s r**
Care workers for the elderly in structure (%)	−0.999	Severe materially deprived—lower secondary education level (%)	0.999

N.B. Only variables correlated with statistical significance at *p* < 0.05.

**Table 4:** Variables correlated to COVID-19 Death Rates on total population (per million people) and on 70+ and 80+ years old (%).

**Effects of Reduction on Total Population Deaths (Per Million)**	**Pearson’s r**	**Effects of Increasing on Total Population Deaths (Per Million)**	**Pearson’s r**
Private Health Expenditure—% of the GDP	−0.999	Two-person poor households (%)	0.999
		Disability Rate—15–64 F (% on 15–64 F)	0.999
		Disability Rate—15+ F (% on 15+ F)	0.999
		Disability rates in activities of daily living (ADL) (%)—55–64 M	0.997
		Disability rates in activities of daily living (ADL) (%)—65+ M	0.999
**Effects of reduction on Death Rate for old (70+) people (on total 70+ pop.) (%)**	**Pearson’s r**	**Effects of increasing on Death Rate for old (70+) people (on total 70+ pop.) (%)**	**Pearson’s r**
% of population with postsecondary education aged 25+ year	−0.998	Dependency Ratio (%)—M	0.999
Active Population—M%	−0.999	Disability rates in activities of daily living (ADL) (%)—55+ M	0.997
**Effects of reduction on Death Rate for old old (80+) people (on total 80+ pop.) (%)**	**Pearson’s r**	**Effects of increasing on Death Rate for old old (80+) people (on total 80+ pop.) (%)**	**Pearson’s r**
% of population with postsecondary education aged 25+ year	−0.999	Dependency Ratio (%)—M	0.999
Active Population—M%	−0.999	Two-person poor households (%)	0.999
		Disability rates in activities of daily living (ADL) (%)—55+ M	0.999

N.B. Only variables correlated with statistical significance at *p* < 0.05.

**Table 5:** Results of the multivariable linear regression models applied to the three countries for Incident Rates (Relative Risk Ratios [RRR]) and constant.

**Dependent Variable: Total Population Cases (per Million); ** **adj. R2 = 0.998.**	***RRR***	***p* < 0.05**
Ageing Index—F	1.65	0.000
Elderly care health facilities rate (% on 65+ pop.)	1.65	0.000
Constant	−398,024.07	0.000
**Dependent Variable: Infection Rate for Old (70+) People (on Total 70+ Pop.) (%);** **adj. R2 = 0.984.**	***RRR***	***p* < 0.05**
Severe materially deprived—Others outside labor force (%)	1.37	0.000
Hospital admission per million	0.50	0.000
Constant	37.24	0.000
**Dependent Variable: Infection Rate for Old Old (80+) People (on Total 80+ Pop.) (%);** **adj. R2 = 0.972.**	***RRR***	***p* < 0.05**
Severe materially deprived—lower secondary education level (%)	8.93	0.000
Care workers for the elderly in structure (%)	3.29	0.000
Constant	−108.16	0.000

Removed variables because of collinearity: Six-and-more persons families; Industry employment (%); Poor persons—All (%); Severe materially deprived—Unemployed (%). Removed variables because of collinearity: Severe materially deprived—Retired (%); Disability Rate—65+ M (% on 65+ M). Removed variables because of collinearity: No variable.

**Table 6:** Results of the multivariable linear regression models applied to the three countries for Death Rates (Relative Risk Ratios [RRR]) and constant.

**Dependent Variable: Total Population Deaths (Per Million); adj. R2 = 0.997.**	***RRR***	***p* < 0.05**
Two-person poor households (%)	1.54	0.000
Private Health Expenditure—% of the GDP	0.57	0.000
Constant	13,298.06	0.000
**Dependent Variable: Death Rate for Old (70+) People (on Total 70+ Pop.) (%); ** **adj. R2 = 0.973.**	***RRR***	***p* < 0.05**
Dependency Ratio (%)—M	2.98	0.000
Disability rates in activities of daily living (ADL) (%)—55+ M	1.10	0.000
Constant	−26.13	0.000
**Dependent Variable: Death Rate for Old Old (80+) People (on Total 80+ Pop.) (%); ** **adj. R2 = 0.968**	***RRR***	***p* < 0.05**
Dependency Ratio (%)—M	1.34	0.000
Disability rates in activities of daily living (ADL) (%)—55+ M	2.04	0.000
Constant	−10.97	0.000

Removed variables because of collinearity: Disability Rate—15–64 F (% on 15–64 F); Disability Rate—15+ F (% on 15+ F); Disability rates in activities of daily living (ADL) (%)—55–64 M; Disability rates in activities of daily living (ADL) (%)—65+ M. Removed variables because of collinearity: % of population with postsecondary education aged 25+ year; Active Population—M%. Removed variables because of collinearity: % of population with postsecondary education aged 25+ year; Active Population—M%; Two-person poor households (%).

**Table 7:** Average values assumed of the covariates left in the regression models for the considered period 2001–2021 by country.

Target Variable	Covariates	Country	Average Value	Sig.
Total population Cases (per Million)	Ageing Index—F	Germany	21.07	n.s.
		Spain	18.6	
		Italy	20.91	
	Elderly care health facilities rate (% on 65+ pop.)	Germany	4.2	*p* < 0.05
		Spain	2.2	
		Italy	3.8	
Infection Rate for old (70+) people (on total 70+ pop.) (%)	Severe materially deprived—Others outside labor force (%)	Germany	6.75	*p* < 0.05
		Spain	5.06	
		Italy	12.36	
	Hospital admission per million	Germany	104,000.0	*p* < 0.05
		Spain	110,616.2	
		Italy	56,913.0	
Infection Rate for old old (80+) people (on total 80+ pop.) (%)	Severe materially deprived—lower secondary education level (%)	Germany	9.03	*p* < 0.05
		Spain	6.42	
		Italy	12.72	
	Care workers for the elderly in structure (%)	Germany	2.2	n.s.
		Spain	2.4	
		Italy	1.9	
Total population Deaths (per Million)	Two-person poor households (%)	Germany	18.16	*p* < 0.05
		Spain	16.41	
		Italy	13.65	
	Private Health Expenditure—% of the GDP	Germany	2.33	n.s.
		Spain	2.25	
		Italy	2.16	
Death Rate for old (70+) people (on total 70+ pop.) (%)	Dependency Ratio (%)—M	Germany	43.07222	n.s.
		Spain	43.79575	
		Italy	45.51052	
	Disability rates in activities of daily living (ADL) (%)—55+ M	Germany	2.8	*p* < 0.05
		Spain	3.3	
		Italy	4.2	
Death Rate for old old (80+) people (on total 80+ pop.) (%)	Dependency Ratio (%)—M	Germany	43.07222	n.s.
		Spain	43.79575	
		Italy	45.51052	
	Disability rates in activities of daily living (ADL) (%)—55+ M	Germany	2.8	*p* < 0.05
		Spain	3.3	
		Italy	4.2	

Legenda: sig. = statistical significance of the differences among countries checked by Pairwise *t*-test (*p* < 0.05). n.s. = no statistical significance; *p* < 0.05 statistical significance.

## Data Availability

The original contributions presented in the study are included in the article. Further inquiries can be directed to the corresponding author.
